# TPEN loaded poly (lactide-co-glycolide) nanoparticles promote neuroprotection and optic nerve regeneration

**DOI:** 10.1016/j.mtbio.2025.101670

**Published:** 2025-03-14

**Authors:** Caiqing Wu, Haitao Zhang, Yuze Chen, Yangjiani Li, Weiwei Jin, Jinpeng Yang, Yehong Zhuo, Ziyu Gao, Xiaohong Hu, Yiqing Li

**Affiliations:** a State Key Laboratory of Ophthalmology, Zhongshan Ophthalmic Center, Sun Yat-sen University, Guangdong Provincial Key Laboratory of Ophthalmology and Visual Science, Guangzhou, 510060, China; b School of Material Engineering, Jinling Institute of Technology, Nanjing, 210000, China; c Institute of Materials in Electrical Engineering 1, RWTH Aachen University, Aachen, 52074, Germany

**Keywords:** N, N, Nʹ, Nʹ-tetrakis-(2-Pyridylmethyl) ethylenediamine (TPEN), Polylactic acid-hydroxyacetic acid (PLGA), Zinc, Nanoparticles, Neuroprotection, Optic nerve regeneration, Glaucoma

## Abstract

Development of novel therapeutics for retinal ganglion cells (RGCs) protection and axon regeneration in neurodegenerative diseases, for example, glaucoma, are critical challenges in clinical treatment. Utilization of N, N, Nʹ, Nʹ-tetrakis-(2-Pyridylmethyl) ethylenediamine (TPEN), a specific chelator of Zn^2+^, revealed positive medical potentials. However, its therapeutic effect in promoting RGCs survival and axon regeneration is restricted due to the inefficient drug delivery and limited absorption. To address this, this work developed a novel nanoparticles (NPs)-based drug delivery system with sustained release of TPEN, using oil-in-water (O/W) single-emulsion solvent evaporation method with various surfaces coatings. Optic nerve crush (ONC) and acute ocular hypertension (AOH) animal models were carried out to investigate the neuroprotective and axon regenerative effects of TPEN-loaded NPs. RGCs protection was systematically assessed through whole-mount retina immunostaining and hematoxylin eosin staining for histological changes. Electroretinography was used for evaluating visual function changes. Axon protection and regeneration were evaluated by SMI32 stain and intravitreal administration of cholera Toxin Subunit B (CTB), respectively. *In vivo*, TPEN-loaded NPs achieved a comparable therapeutic effect on neuroprotection and axon regeneration after ONC, with a reduced frequency of vitreous injection and half of TPEN dosage compared to its solution. In the meantime, visual function was also more effectively preserved with TPEN-loaded NPs. Additionally, RGCs survival and axon protection after AOH were significantly enhanced after treating with TPEN-loaded NPs. The developed TPEN-loaded NPs show promise for pre-clinical testing of neuroprotective and neuro-regenerative therapies in glaucoma and other neurodegenerative diseases.

## Highlights

• Novel TPEN-loaded NPs with sustained release were developed via O/W single-emulsion solvent evaporation and surface coatings.
• Increased TPEN injection frequency boosts neuroprotection & axon regeneration post-ONC injury.
• Single TPEN-NP dose (0.127μg) outperforms equal solution dose, matches double solution (0.254 μg) in ONC recovery.
• TPEN-NPs (0.127 μg) show superior RGC/axon protection vs solution in acute ocular hypertension model.

## Graphical abstract

TPEN-loaded nanoparticles fabricated by single emulsion solvent evaporation method with oil-in-water (O/W) promoted RGCs survival and axon regeneration in glaucomatous animal models.

**Figure:**
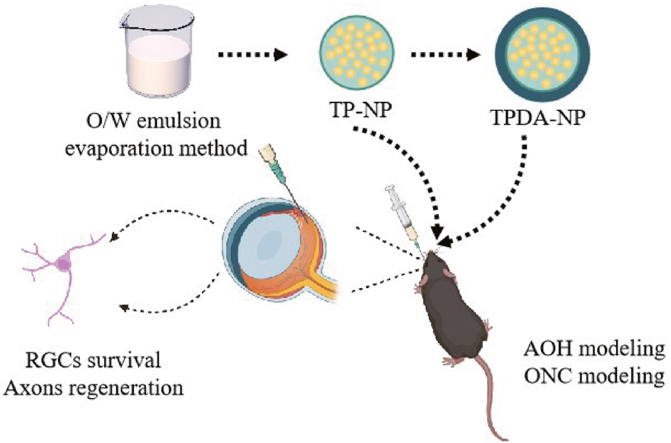
TPEN-loaded nanoparticles fabricated by single emulsion solvent evaporation method with oil-in-water (O/W) promoted RGCs survival and axon regeneration in glaucomatous animal models.

## 1 Introduction

Glaucoma, an irreversible optic neuropathy, has been a leading cause of blindness globally [1]. According to statistics, around 111.8 million glaucoma cases are estimated worldwide by 2040 [2]. Noticeably, the loss of retinal ganglion cells (RGCs) and progressive damage to their axons, which lack regenerative abilities in adult mammals are characterized as the key features of glaucoma [3]. Numerous endeavors including manipulations of signal transduction pathways, modulation of transcription factors, and cell-extrinsic suppressors of axon growth, have been studied to protect RGCs from death and to facilitate RGC axon regeneration, however, effective clinical treatments for optic nerve damage remain elusive [[4], [5], [6], [7], [8]].

Despite various underlying mechanistic pathways, zinc (Zn), as an essential trace element in all living organisms [9], appeared critical effect in promoting RGCs survival and maintaining optic functions of retina [10]. In neurons, about 90 % of the total zinc are dominantly bound to metalloenzymes, transcription factors, and other zinc-containing proteins, while the remaining 10 % either exists as hydrated ions or weakly bind to proteins as mobile zinc (Zn^2+^) [11,12]. In our previous work, the influence of Zn^2+^ dysregulation after optic nerve crush was systematically studied and reported that Zn^2+^ accumulation could pivotally initiate the death of RGCs in retinas [10]. Downregulation of Zn^2+^ in retinas can be achieved either genetically, for example, through conditional knockout of ZnT3 in amacrine cells [13]; or pharmacologically, such as using the Zn^2+^ specific chelator N, N, Nʹ, Nʹ-tetrakis-(2-Pyridylmethyl) ethylenediamine (TPEN) [14]. Although both of these strategies also demonstrated promising ability in safeguarding RGCs and promoting axons regeneration, the development of innovative therapeutic approaches to address diseases associated with optic nerve damage remains a critical challenge in clinical settings [4,14,15].

Given consideration to the clinical accessibility and availability, great attention has been paid to pharmacological solutions, e.g. TPEN, as a novel drug for neuron protection [10,13,14]. However, the application and translation of TPEN in treating optic neuropathy has been severely constrained due to the inefficient drug transportation and low absorption *in vivo*, as a result of the hydrophobic nature of TPEN molecule itself. To enhance the therapeutic efficacy in regulating Zn^2+^ related optic neuropathies, the investigation and development of a sustained delivery system for TPEN emerge as crucial endeavors.

To better design and formulate drug delivery systems, nanomaterials, particularly, nanoparticles (NPs) have been widely used due to their highly effective surface area at nanoscale and high permeability for *in vivo* drug transportation [15,16]. Pharmaceutical polymers that approved by the US Food and Drug Administration (FDA), for example, polylactic acid-hydroxyacetic acid (PLGA), are drawing arising attention as drug carriers. Additional to the well-known contribution in biodegradability and biosafety, PLGA also demonstrated versatility in drug encapsulation, structure formulation and chemistry functionalization at nanoscale for effective biomedicine purpose [17]. However, only about 20 products utilizing PLGA have been approved by the FDA since 1989 due to the initial burst drug release in many PLGA drug-carrying systems both *in vitro* and *in vivo*, underscoring the ongoing challenges in developing sustained-release formulations for PLGA drug carriers [18].

Integrate adhesive and biocompatible components with PLGA based systems revealed effectiveness in mitigating the initial drug abruption and sustaining the whole release process [19]. For example, significant achievement was reported by using polydopamine (PDA) coatings to regulating paclitaxel transportation from PLGA carrier [20]. As a bioinspired material from mussel, PDA provides a biomimetic system with the maintained adhesive and biocompatible natures. Furthermore, it offers a simple preparation process with the ease of bio functionalization by surface coating [21], gives a window towards a sustainable delivery and effective therapeutic system [20].

This work therefore focused on developing a novel nanoparticle-based drug carrier for TPEN encapsulation and transportation, by introducing biocompatible PLGA and DPA to facilitate the clinical operation and translation. Here, we report our progress in fabrication and functionalization of TPEN-loaded particles, utilization of TPEN release property both *in vitro* and *in vivo*. Particularly, two animal models were introduced for *in vivo* evaluation in this work, 1) optic nerve crush (ONC) models, whom has been widely used in neuronal survival and axonal regeneration study within the central nervous system due to their accessibility, anatomic and functional importance, *etc*. [10]; and 2) acute ocular hypertension (AOH) induction model, whom has been extensively employed in glaucoma studies, given the heightened intraocular pressure (IOP) as one of the noticeable features of the disease [22]. The influence of TPEN and TPEN-loaded nanoparticles on RGCs protection, axon regeneration and visual function recovery were systematically evaluated and reported with both ONC and AOH animal models.

## 2 Materials and methods

### 2.1 Materials

Different molecular weight (10 kDa, 30 kDa, 32 kDa, 48 kDa) of polylactic acid-hydroxyacetic acid (PLGA, CAS: 34346-01-5) were purchased from Jinan Daigang BIO Engineer Limited Co.Ltd., China. Polyvinyl alcohol (PVA, CAS: 9002-89-5) was obtained from Shanghai Sinophram Chemical Reagent Co.Ltd., China. Dopamine hydrochloride (DA, CAS: 62-31-7) and dichloromethane (DCM, CAS: 75-09-2) were purchased from Adamas. N,N,Nʹ,Nʹ-tetrakis-(2-Pyridylmethyl)ethylenediamine (TPEN, CAS: 16858-02-9) was from Merck Millipore (6163941, Germany). All other reagents and solvents were purchased from Sigma-Aldrich (Missouri, the USA) of analytical grade and used as received.

### 2.2 Development of TPEN-loaded NPs

#### 2.2.1 Fabrication of TPEN-loaded PLGA NPs

TPEN-loaded PLGA NPs were fabricated by oil-in-water (O/W) single emulsion solvent evaporation method. The TPEN and different molecular weight (10 kDa, 30 kDa, 32 kDa, 48 kDa) of PLGA were add to the DCM, then mixed with a vortex mixer for 10 min to make them completely dissolved. After the addition of a certain amount of PVA aqueous solution (0.3 %, v/v), the mixture was then rapidly emulsified through a microprobe sonication (900W power) for 1 min. The emulsion was subsequently transferred into deionised water and stirred at 1400 rpm for 2h to evaporate the organic solvent. Afterwards, the TPEN-loaded PLGA NPs were collected by centrifugation (14,000 rpm, 5 min), followed with three-time wash and re-centrifuge cycles with deionised water. The TPEN-loaded PLGA NPs collected by centrifugation were lyophilized for subsequent experiments.

#### 2.2.2 Modification of NPs formation

100 mg of TPEN-loaded PLGA NPs were redispersed in 10 mL Tris buffer (10 mM, pH = 8.5) with subsequent addition of 10 mg DA. DA is formed as a PDA coating on the surface of the PLGA nanoparticles through covalent bonding and non-covalent self-assembly during the oxidation process by reacting under magnetic stirring in an ice-water bath for 3h. The PDA coated NPs were obtained by centrifugation at 14,000 rpm for 5 min. In summary, the following NPs were formulated:

P-NP: PLGA NPs.

PDA-NP: Polydopamine-coated PLGA NPs.

TP-NP: TPEN-loaded PLGA NPs.

TPDA-NP: TPEN-loaded polydopamine-coated PLGA NPs.

#### 2.2.3 Particle size, morphology and composition characterization of NPs

To evaluate the particle size of the NPs, nanocrystalline particles were dispersed uniformly in water using microprobe sonicator, at 900W power (JY92-ⅡDN, SCIENTZ, China) for 1 min. The NPs suspension was diluted 1/100 (v/v) in distilled water and analyzed using dynamic light scattering (Zetasizer Nano ZEN3600, Malvern, UK). For the measuring methods, the materials were characterized as polystyrene emulsions, and measurements were conducted at 25 °C with 300-s equilibration time and 90 °test angle. Three sets of particle size measurement were carried out with 15 rounds per set.

To investigate the NPs morphology, scanning electron microscopy (SEM, GeminiSEM300, ZEISS, Germany) was employed, operated at a low accelerating voltage of 5 kV. To prepare conductive specimens, thin layers of a gold-plated palladium alloy were sputtered onto the samples using a HITACHI MC1000 ion sputter. Moreover, the surface elemental composition of the NPs was analyzed using a Thermo Fisher ESCALAB 250Xi X-ray photoelectron spectrometer, focusing on monochromatic Al-Kα radiation.

#### 2.2.4 TPEN loading and releasing behavior of NPs *in vitro*

The drug loading in TP-NP was determined by UV–Vis spectrophotometry (BioSpectrometerbasic, Eppendorf, Germany). After collecting the TP-NPs by centrifugation, the absorbance of the centrifuged supernatant at 260 nm was measured by UV–Vis spectrophotometer. The amount of free TPEN in the supernatant was calculated from the measured TPEN standard curve and the total volume of the solution. The drug loading (LC%) of TP-NP was calculated by Equation (1):

$$\begin{array}{c} LC\%=\frac{Total\;amount\;of\;TPEN-amount\;of\;\text{free}\;TPEN\;in\;the\;supernatant}{Total\;weight\;of\;TP-NP\;after\;lyophilisation}\left(\;\text{AUTONUM}\;\backslash *\;\text{Arabic}\;\right) \end{array}$$

To evaluate the TPEN releasing behavior *in vitro*, the NPs loaded with TPEN were resuspended in 5 mL of PBS buffer (10 mM phosphate, pH 7.4) and oscillated in an air bath shaker at 37 °C. At regular time points (1h, 4h, 24h, 48h, 96h, 192h), the NPs suspension was centrifuged at 14,000 rpm for 3 min, and 3 mL of the supernatant was collected and replaced with fresh buffer. The remaining NPs were collected by centrifugation and re-dispersed in ethanol. The concentration of TPEN in the collected supernatant samples and the remaining NPs was analyzed by UV–Vis spectrophotometer. And the cumulative release amount of TPEN is calculated by Equation (2):

$$\begin{array}{c} M_{n}=C_{n}\times 5+C_{n-1}\times 2+C_{n-2}\times 2+\ldots \ldots +C_{3}\times 2+C_{2}\times 2+C_{1}\times 2\;\left(\;\text{AUTONUM}\;\backslash *\;\text{Arabic}\;\right) \end{array}$$

Where:

M is the cumulative release amount of TPEN,

C is the concentration of TPEN in solution,

n is the ordinal number of samples.

### 2.3 Cytotoxicity test

Human retinal pigment epithelial (RPE) cell line: the aRPE-19 cells (American Type Culture Collection, the USA) and the BV2 cell line (Zhong Qiao Xin Zhou Biotechnology Company, Shanghai, China) were cultured in Dulbecco's Modified Eagle Medium/Nutrient Mixture F-12 (DMEM/F-12, Gibco, Thermo Fisher Scientific, the USA) culture medium. Murine photoreceptor cell line: the 661W cells generously provided by Dr. Yuxun Shi (State Key Laboratory of Ophthalmology, Zhongshan Ophthalmic Center, Sun Yat-sen University, Guangdong Provincial Key Laboratory of Ophthalmology and Visual Science) were routinely cultured in DMEM high glucose medium (Gibco, Thermo Fisher Scientific, the USA). A total of 10 % fetal bovine serum (FBS, Gibco, Thermo Fisher Scientific, the USA) and 1 % penicillin and streptomycin (HyClone, GE Healthcare Life Science, the USA) were added into the two culture mediums listed above. All kinds of cells were cultured in a 5 % CO_2_ humidified environment at 37 °C (Thermo Fisher Scientific, the USA), and treated with 0, 1 μM, 10 μM, 100 μM, 1 mM, and 10 mM of TPEN solution for 24 h to test the impact of concentration of TPEN on cell viability. TPEN-loaded NPs suspension containing 1 μM or 10 μM TPEN were also cultured for 24h to test the cell viability. The culture medium was extracted and washed twice with PBS. The cells were than incubated with 100ul medium containing 10ul Cell Counting Kit-8 (CCK-8, Dojindo, Japan) reagent for 50 min at 37 °C and absorbance was measured at a wavelength of 450 nm. Cell survival rate of every plate was used for estimating cytotoxicity by CCK-8 Kit.

### 2.4 Experiments *in vivo*

#### 2.4.1 Animal maintenance and operation

All animal experiments were strictly complied with the principal of Association for research in vision and ophthalmology (ARVO) statements and were conducted with approval from the ethics committee of animals in Zhongshan ophthalmic center (SYXK2018-0189) at Sun Yat-Sen University (IACUA: Z2022071). Wild-type C57BL/6J mice of both genders, aged between 6 and 8 weeks, were used in this study. The mice were housed in standard cages in a specific pathogen-free facility with a 12-h light/dark cycle with free access to food and water. Mice were randomly assigned for each experiment after 7-day acclimation.

Surgeries of optic nerve crush (ONC) were carried out with established protocols [10]. Briefly, mice were general anesthetized by intraperitoneal injection of pentobarbital sodium (100 mg/kg), and an incision was applied at the superior side of the mice conjunctiva and retrobulbar optic nerve (ON). Using forceps (RWD Life Science Co., Ltd., China), the optic nerve (ON) was then crushed approximately 2 mm posterior to the globe for 5 s. The acute ocular hypertension (AOH) animal model was established as previously reported [23]. Pupils of experimental mice were dilated by 1 % compound tropicamide (Alcon, Switzerland) after been fully anesthetized by intraperitoneal injection of pentobarbital sodium (100 mg/kg). Then, the anterior chamber of the right eye was cannulated with a 30-gauge needle (LOUNBIOTECH, China) attached to an infusion tube, allowing for 1-h perfusion of sterile 0.9 % saline solution. TPEN solution (100 μM) or TPEN loaded NPs suspension were administered by intravitreal injection (3 μL per eye) after ONC or AOH immediately [10,24,25]. The antibiotic ophthalmic ointment was applied to surgical eyes as post-surgery or post-intraocular injection care. Finally, the mice were resuscitated on a warm pad (Yancheng, China).

#### 2.4.2 Hematoxylin and eosin staining

Eyes designated for hematoxylin and eosin staining (HE) staining were collected 7 days post-treatment or 14 days post-injury. After cardiac perfusion with 4 % PFA, eyeballs were carefully extracted and fixed overnight at 4 °C. The eyeballs were subsequently embedded in paraffin and sectioned into 5 μm slices through the optic disc after ethanol dehydration. Then, the slices were stained with hematoxylin and eosin and imaged with Pannoramic MIDI scanner (3DHISTECH, Hungary). The thickness of whole retina, retinal nerve fiber layer (RNFL), ganglion cell layer (GCL), inner plexiform layer (IPL), inner nuclear layer (INL), outer plexiform layer (OPL), outer nuclear layer (ONL) and ganglion cell complex (GCC) in retinas were analyzed by ImageJ software. Retinal cell densities in GCL, INL, and ONL were quantified by ImageJ software.

#### 2.4.3 Zn^2+^ autometallography

Autometallography was performed on day 1 post-ONC as described previously [10]. Briefly, Sodium selenite (1.5 mg/mL in distilled water, Sigma, China, 15 mg/kg) was injected intraperitoneally 2h before mice suffered transcardial perfusion with isotonic saline (Sigma) and 2.5 % (v/v) glutaraldehyde (ED-8484, ECOTOP, China) in 0.1 M phosphate buffer (pH, 7.4) under inhalation anesthesia with isoflurane (3 % for 2 min, RWD Life Science Co., Ltd., China). Eyes were removed and fixed in 2.5 % glutaraldehyde at 4 °C for 2h. The tissues were cryoprotected in 30 % sucrose at 4 °C overnight, cryosectioned at 14 μm, and mounted on coated slides. For AMG staining, slides were air-dried, fixed in 95 % ethanol for 15 min, rehydrated in 70 % and 50 % ethanol, and rinsed in distilled water before being dipped in 0.5 % gelatin solution (9000-70-8, Macklin, China). Slides were then developed in AMG developer solution for 3.5h in the dark at 150 rpm. The AMG developer consists of 30 mL gum Arabic (V900768-500G, Sigma-Aldrich, the USA), 5 mL citrate buffer (C2404, Sigma-Aldrich, the USA), 0.425g hydroquinone (H9003-500G, Sigma-Aldrich, the USA) in 7.5 mL distilled water, and 0.06 g silver-lactate (359750-5G, Sigma-Aldrich, the USA) in 7.5 mL distilled water. Then the slides were washed in 37 °C running water for 10 min, treated with 5 % sodium thiosulfate for 12 min, rinsed in distilled water, post-fixed in 70 % ethanol, and sequentially dehydrated in 95 % ethanol, 100 % ethanol, and xylene (1330-20-7, Macklin, China) before being mounted with xylene-based mounting medium and coverslips. At least three images from different areas of each retinal section were captured under bright-field illumination (DMi8, Leica, Germany). The intensity of the Zn^2+^-autometallography signal in the inner plexiform layer (IPL) of retinas was analyzed using Image J software (the USA).

#### 2.4.4 Retinal flat-mount immunofluorescence

Mice underwent perfusion fixation through heart using isotonic saline with 4 % (w/v) paraformaldehyde (PFA, Biosharp, China) after 2 weeks post-surgeries. Subsequently, eyes of mice were collected and immersed in 4 % PFA for an additional 2h with corneal incisions. The retinas were then dissected and subject to overnight incubation with βIII-tubulin antibodies (801202,1:1000, Biolegend) and RNA-binding protein with multiple splicing (RBPMS, GTX118619, 1:1000, GeneTex) at 4 °C. Following with 2-h incubation of Alexa Fluor 488-conjugated secondary antibody to mouse IgG (ab150105, 1:500, Abcam) and 555-conjugated secondary antibody to rabbit IgG (ab150074, 1:500, Abcam). The whole retinas were divided into four parts and flattened for photography. The central, middle and peripheral areas for each part were imaged with light microscopy (DMi8, Leica, Germany). ImageJ software was employed to count both βIII tubulin and RBPMS positive cells from 12 images per retina. The number of both βIII tubulin and RBPMS positive cells per mm^2^ was determined and divided by the field area. The final average number of RGCs per retina was calculated from 12 images.

#### 2.4.5 Quantitation of regenerated axons in the optic nerve

The quantification of regenerated axons after ONC was conducted following previous established protocols [10]. Cholera Toxin Subunit B (CTB, C22843, ThermoFisher) was administrated via intravitreal injection 3 days before mice were euthanized for axon regeneration assessment. Mice were perfused and fixed by isotonic saline and 4 % (w/v) PFA through heart after 2 weeks of ONC. The CTB-labeled optic nerves of mice were collected and immersed in 4 % PFA for an additional 2h and then placed in 30 % sucrose for at least 6h. Longitudinal cryosections (14 μm) of optic nerves were obtained using a cryostat (CM1950, Leica, Germany). The quantification of CTB-labeled regenerated axons, extending from the injury site at various distances, were examined under 400× magnification of light microscopy (DMi8, Leica, Germany), and at least 4 cross sections per sample was calculated. The number of CTB-labeled axons were normalized to the cross-sectional area of the optic nerve and extrapolated to represent the entire optic nerve.

#### 2.4.6 UPLC-ESI-MS/MS analysis

To assess TPEN release in retinas, an ultra-high-performance liquid chromatography tandem mass spectrometry (UPLC-ESI-MS/MS) system (ACQUITY UPLC, Waters, the USA) was utilized. In this work, all the retinal samples were stored at 4 °C prior to analysis, maintaining uniform LC-MS condition for sample measurement. 5 μL of extractive retinal samples were used to detect TPEN. The mobile phase was consisted of solvent A (25 mM ammonium acetate and 25 mM ammonia monohydrate in H_2_O) and solvent B (Acetonitrile). Separation of TPEN was achieved using an ACQUITY UPLC® BEH C18 column (1.7 μm, 2.1 × 100 mm, Waters, Milford, MA) maintained at 40 °C, with a flow rate of 0.3 mL/min. Detection of the TPEN in retinal samples was performed using both a Diode array detector (DAD) and a mass spectrometer (AB sciex, 5600 Q-TOF/6500 Q-TRAP).

The effluent was alternatively directed to an electrospray ionization-triple quadrupole-linear ion trap (ESI-QTRAP)-MS system. The ESI source operated with the following parameters: source temperature at 500 °C; ion spray voltage (IS) at 5500 V (positive ion mode)/-4500 V (negative ion mode); ion source gas I (GSI), gas II (GSII), and curtain gas (CUR) set at 50, 60, and 25 psi, respectively. Collision-activated dissociation (CAD) was optimized to high setting. Instrument tuning and mass calibration utilized 10 and 100 μmol/L polypropylene glycol solutions in QQQ and LIT modes, respectively. QQQ scans were acquired as multiple reaction monitoring (MRM) experiments with collision gas (nitrogen) set to medium. Declustering potential (DP) and collision energy (CE) for individual MRM transitions were optimized with further necessary adjustments. A specific set of MRM transitions were monitored for each period based on the elution of metabolites within respective timeframes.

#### 2.4.7 *In vivo* visual function evaluation

Pattern electroretinogram (pERG) of mice was recorded by a Retiport system (Roland Consult, Brandenburg, Germany). Experiments were performed at 14 days of pre- and post-AOH injury. Mice were anesthetized as previously described and the pupils were dilated using compound tropicamide (Alcon). During the pERG experiments, three electrodes (Roland Consult, Brandenburg, Germany) were applied: an active ring-shape electrode was placed in contact with the cornea, while the needle electrodes served as the reference and ground electrodes, positioned subcutaneously in the face and tail, respectively. The responses were evoked by an alternating and black-and-white horizontal grating pattern displayed on a screen approximately 18 cm away from the central corneas. The superposition of 100 successive fringe lattice reactions constituted pERG waveform, with two successive waveform captures recorded for each experiment. The P1-N2amplitude, from the peak of the P1 wave to the trough of the N2wave, was measured [26].

The flash visual-evoked potential (f-VEP) (RETI-scan21, Roland Consult, Brandenburg, Germany) was also conducted 2 weeks of pre- and post-AOH injury. Three needle electrodes Roland Consult, Brandenburg, Germany) were used for f-VEP recording: placed in the occipital region of the skull (active electrode), the forehead (reference electrode) and tail (ground electrode). The waveform, the superposition of 80 flash stimuli at 1.0 Hz, was recorded, and three successive waveforms were recorded for each individual experiment. The N2-P2 amplitude was measured [27].

#### 2.4.8 Immunofluorescence staining

Following with heart perfusion and fixation, the optic nerves of mice were harvested and post-fixed on ice for 2h, dehydrated overnight with 30 % sucrose in 0.01 M PBS until sinking, then embedded in OCT (4583, SAKURA). Transverse cryosections (10 μm) were performed using a cryostat (CM1950, Leica, Germany) and stored at −80 °C refrigerator (BD-80, Meiling, China) until use. Proximal (a distance of 0.6–0.8 μm behind the globe), middle (the middle of the optic nerve), and distal (a distance of 0.6–0.8 μm ahead of the optic chiasm) sites were dissected. All sections were mounted on slides and stored at 20 °C. To obtain a representative sampling, consecutive sections were cut, and each 5 slides were collected for staining. At least three sections were analyzed per case. Sections were blocked with normal donkey serum (ASL050, Acmec biochemical) and incubated overnight at 4 °C with primary antibodies targeting SMI32 (SMI-32P, 1:500, Biolegend). After washing, the sections were then incubated with fluorescent secondary antibodies (1:500, Abcam, Thermo Fisher Scientific) for 1h at room temperature. Finally, the slides were covered with a coverslip. Immunofluorescent staining images were captured with a microscope (LSM980, Zeiss, Germany). The total average count of SMI32^+^ stained axons in proximal, middle and distal sites was analyzed.

### 2.5 Statistical analysis

Experiments were performed at least three times in each group in this study. Data were analyzed in a blind manner. Each point on the statistical chart represents an individual data point. Data are presented as mean ± standard error of the mean (SEM). Differences between groups were conducted using a one-way analysis of variance followed by Dunnett's multiple comparison test or Student's t-test (2-tailed) by Prism 8 (GraphPad, the USA). A p-value of less than 0.05 represented statistically difference. Detailed descriptions of all statistical analyses, including the tests employed, p-values, and exact sample sizes (n values), are provided in the figures and manuscript.

## 3 Results and discussion

### 3.1 Synthesis and characterization of NPs

**Fig. 1:**
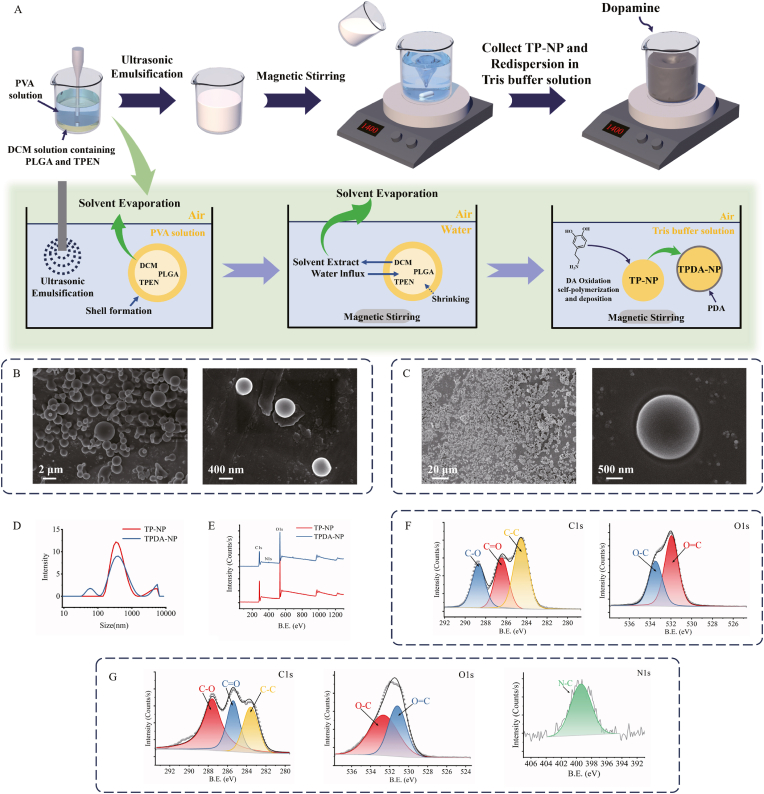
**Characteristics of NPs. (A)** Schematic of the formation of TPEN-loaded NPs. (**B**) SEM image of TP-NP. (**C**) SEM image of TPDA-NP. (**D**) Particle size distribution of TP-NP and TPDA-NP. (**E**) Survey XPS spectra of TP-NP and TPDA-NP. (**F**) High-resolution core-level XPS spectra of TP-NP. (**G**) High-resolution core-level XPS spectra of TPDA-NP.

TP-NP were prepared using the two-step O/W emulsion solvent evaporation method to address low drug entrapment efficiency that induced by TPEN solubility issue [28,29], following with oxidation-induced DA polymerization to obtain TPDA-NP, as illustrated in Fig. 1A. In detail, the TPEN and PLGA were dissolved in DCM (1 mL) as the oil phase, which was then dispersed into PVA/H_2_O solution as the aqueous phase and emulsified under an ultrasonic microprobe. Here, the usage of water phase during emulsification was reduced to mitigate the drug loss and decrease the surfactant quantity to minimize bio risks. The DCM volatilization was initiated by the thermal effect during ultrasonic emulsification. Initial shells of nanoparticles were formed following with PLGA molecules agglomeration, which were subsequently dispersed in a large amount of deionised water and stirred to completely volatilize the DCM. The shells then shrank, forming local and dense microstructure surface around the TPEN-PLGA particles [30]. The solidified TP-NPs were introduced into a dopamine solution, which was oxidized and self-polymerized to PDA under alkaline condition. The solution color change from white to dark gray indicated successful DA polymerization, forming adhesive PDA layer to encapsulate TP-NPs, the initial burst release of TPEN shall be reduced by this nanocoating.

The morphologies of TP-NP (Fig. 1B) and TPDA-NP (Fig. 1C) were observed by SEM. Both TP-NP and TPDA-NP appeared regular spherical shapes with smooth surfaces and no obvious pores. Changes in particle size before and after modification were assessed using dynamic light scattering, which can effectively detect the overall particle size and distribution. In comparison with TP-NP, the particle size of TPDA-NP increased from 421 ± 3 nm (PDI = 0.25 ± 0.02) to 467 ± 8 nm (PDI = 0.53 ± 0.13) (Fig. 1D). Surface composition analysis of TP-NP and TPDA-NP was conducted by X-ray photoelectron spectroscopy (XPS) (Fig. 1E–G). In the TPDA-NP spectrum, a distinctive N-C peak of N1s was observed at 399.6 eV, signifying the successful coating of PDA on the surface of PLGA nanoparticles. The percentages of different elements before and after modification are listed in Table 1.

**Table 1:** The percentage of different elements.

	C	O	N
TP-NP	63.14 %	36.86 %	–
TPDA-NP	60.64 %	37.80 %	1.56 %

### 3.2 Optimization of TP-NP fabrication

Table 2 demonstrates the encapsulation rate and particle size of the synthesized TP-NP with varying PLGA molecular weights, O/W ratios and PLGA concentrations. The particle size exhibited a significant increase along with the PLGA molecular weight from 315 ± 2 nm (10 kDa) to 935.1 ± 15 nm (48 kDa). The heightened viscosity of the organic phase resulted in the formation of larger droplets during sonication, contributing to an augmentation in particle size [31]. Unexpectedly, the encapsulation efficiency of TPEN was unaffected across all PLGA molecular weights, despite the increase in PLGA molecular weight leads to a longer diffusion path from the organic phase to the aqueous phase and could potentially reduce drug loss. This resilience can be attributed to the water solubility of TPEN.

**Table 2:** Preparation parameters, encapsulation efficiency and particle size of TP NP prepared by O/W emulsion method.

PLGA MW (kDa)	PLGA Concentration(mg/mL)	O/W ratio	Encapsulation efficiency (%)	Particle size (nm)	PDI
10	40	1:3	50.4 ± 1.5	315 ± 2	0.20 ± 0.01
32	40	1:3	51.0 ± 2.2	636 ± 13	0.32 ± 0.03
48	40	1:3	49.8 ± 2.3	935 ± 15	0.23 ± 0.05
30	40	1:2	43.4 ± 2.7	710 ± 2	0.43 ± 0.01
30	40	1:3	49.5 ± 1.2	572 ± 2	0.31 ± 0.02
30	40	1:4	38.3 ± 3.8	382 ± 1	0.23 ± 0.01
30	40	1:8	28.0 ± 3.7	332 ± 1	0.13 ± 0.02
30	40	1:3	49.5 ± 1.9	572 ± 13	0.27 ± 0.03
30	60	1:3	46.7 ± 2.1	653 ± 20	0.26 ± 0.03
30	80	1:3	48.6 ± 2.4	654 ± 6	0.33 ± 0.01
30	100	1:3	47.7 ± 1.7	698 ± 2	0.15 ± 0.03

Reduction in particle size was observed while decreasing the O/W ratio from 1:2 (710 ± 2 nm) to 1:8 (332 ± 1 nm), respectively, which corresponded to an increase of the aqueous phase. The expansion of aqueous volume introduced more PVA to disperse at the organic and aqueous interface. This dispersion reduces interfacial tension, preventing droplets from aggregation and, consequently, reducing particle sizes. Furthermore, post remove the organic solvent allowed additional PVA molecules to physically bind to the particle surface. The abundant hydroxyl groups on PVA extended into the continuous phase to form a hydrated layer, preventing particle agglomeration. As the O/W ratio decreases, the encapsulation of TPEN initially increased slightly from 43.4 ± 2.7 % (1:2) to 49.5 ± 1.2 % (1:3), the initial TPEN diffusion in the aqueous phase was slightly hidden due to the PVA dispersion at the emulsion surface [32]. However, when the aqueous phase increased to 1:8, the distribution of TPEN in the organic phase decreases, leading to a rapid reduction in the encapsulation rate of TPEN to 28.0 ± 3.7 %.

The particle size increased from 572 ± 13 nm (40 mg/mL) to 698 ± 2 nm (100 mg/mL) with increase in PLGA concentration, similar with the influence of molecular weight. The viscosity of the organic phase increases with PLGA concentration, thereby diminishing net shear stress. This might impede the organic phase disperse into aqueous phase, generating larger droplets and subsequently larger particles post-organic solvent removal [33]. Furthermore, as PLGA concentration increases, PVA might prove insufficient to cover entire droplet surfaces, leading to droplet aggregation during organic solvent evaporation. In the other hand, the surge in PLGA concentration augments the viscosity of the organic phase, intensifying resistance to drug diffusion, therefore could potentially enhance the encapsulation rate [33]. However, the encapsulation rate of TPEN exhibited slightly decrease but not significant change from 49.5 ± 1.9 % to 47.7 ± 1.7 % while increasing PLGA concentration from 40 to 100 mg/mL, respectively. Since TPEN demonstrates solubility in water, diffusion into the aqueous phase during the emulsification of the organic phase with the aqueous phase leads to difficulties in further improving the encapsulation rate of the TPEN [28,29].

### 3.3 Drug release behavior of TP-NP and TPDA-NP *in vitro*

**Fig. 2:**
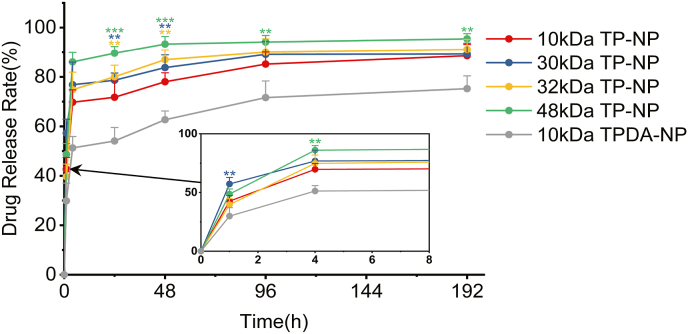
**Effect of different PLGA molecular weight on drug release rate in vitro.** The data were analyzed by one-way ANOVA with Bonferroni’s post hoc test. Green ∗, blue ∗ and yellow ∗ mean the difference between groups of TP-NP (molecular weight of PLGA was 48 kDa), TP-NP (molecular weight of PLGA was 30 kDa), TP-NP (molecular weight of PLGA was 32 kDa) and TPDA-NP (molecular weight of PLGA was 48 kDa 10 kDa), respectively. ∗∗P＜0.01, ∗∗∗P＜0.001 between indicated two groups. (For interpretation of the references to color in this figure legend, the reader is referred to the web version of this article.)

The *in vitro* drug release of TP-NP and TPDA-NP with different compositions were examined, exhibiting roughly three-stage profiles as depicted in Fig. 2. The release rate of TPEN from TP-NP surpassed 90 % during the 240-h evaluation. A pronounced initial burst release of TPEN was observed during the first 4h in all NP cases. The rest of TPEN in subsequent stages was completely released along with PLGA degradation [34]. As the TP-NPs sizes decrease along with the PLGA molecular weight, the effective surface area of the particles increased, resulting in an augmented amount of adsorbed PVA, which could subsequently retard the release of TPEN [35]. However, significantly reduction of TPEN release was achieved in the case of PDA encapsulated NPs, lowering to 51 % in the first 4h and gradually reaching 82 % in the ensuing 240h. In addition, the significant difference between the TPDA-NP and other groups (P = 0.009 VS. 30 kDa TP-NP group, 1h; P = 0.003 VS. 48 kDa TP-NP group, 4h; P = 0.009 VS. 30 kDa TP-NP group, 24h; P = 0.006 VS. 32 kDa TP-NP group, 24h; P = 0.0007 VS. 48 kDa TP-NP group, 24h; P = 0.006 VS. 30 kDa TP-NP group, 48h; P = 0.003 VS. 32 kDa TP-NP group, 48h; P = 0.0005 VS. 48 kDa TP-NP group, 48h; P = 0.009 VS. 48 kDa TP-NP group, 96h; P = 0.009 VS. 48 kDa TP-NP group, 192h) further indicated the retarded release of TPEN.

### 3.4 Toxicity test *in vitro* and *in vivo*

**Fig. 3:**
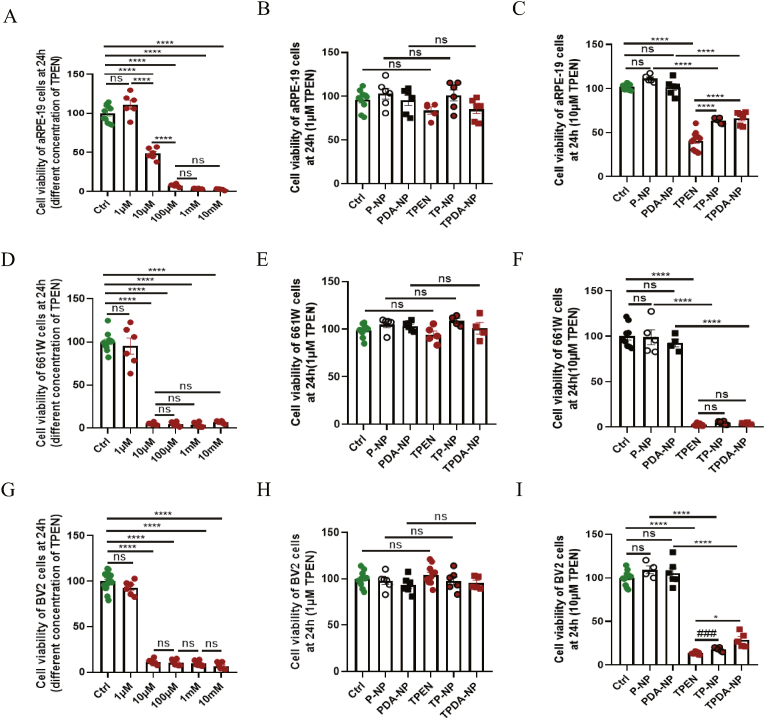
**Toxicity of TPEN-loaded nanoparticles in vitro.** (**A**) Cell viability of aRPE-19 cells treated with different concentration of TPEN solution after 24 h. (**B**) Cell viability of aRPE-19 cells in different groups containing 1 μM TPEN. (**C**) Cell viability of aRPE-19 cells in different groups containing 10 μM TPEN. (**D**) Cell viability of 661W cells treated with different concentration of TPEN solution after 24 h. (**E**) Cell viability of 661W cells in different groups containing 1 μM TPEN. (**F**) Cell viability of 661W cells in different groups containing 10 μM TPEN. (**G**) Cell viability of BV2 cells treated with different concentration of TPEN solution after 24 h. (**H**) Cell viability of BV2 cells in different groups containing 1 μM TPEN. (**I**) Cell viability of BV2 cells in different groups containing 10 μM TPEN. The data were analyzed by one-way ANOVA with Bonferroni’s post hoc test and unpaired Student’s t-test (2 tailed). ∗P＜0.05, ∗∗∗∗P＜0.0001 between indicated two groups. ###P＜0.001 between indicated two groups.# means unpaired Student’s t-test (2 tailed) between indicated two groups. ns, no significant difference in indicated two groups. Each point shown in the bar charts indicates an individual data point.

**Fig. 4:**
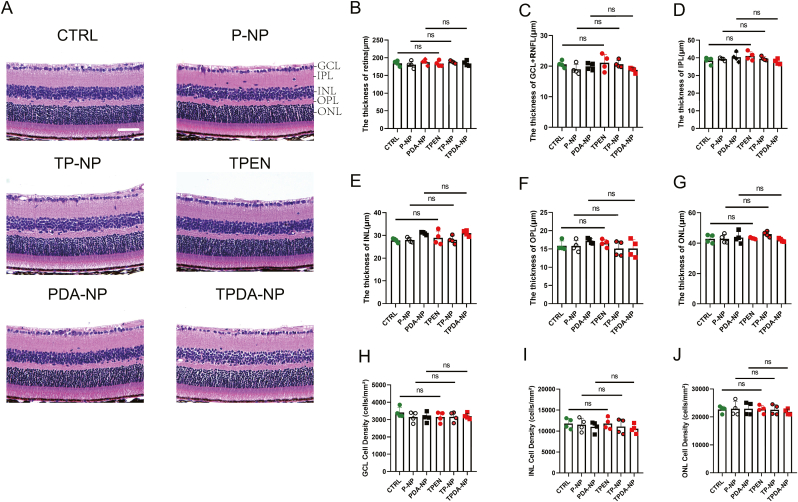
**Toxicity of TPEN-loaded nanoparticles in-vivo.** (**A**) Representative images of hematoxylin and eosin staining in different groups. Scale bar, 50 μm. (B-G) The thickness of retina (**B**), GCL+NFL (**C**), IPL (**D**), INL (**E**), OPL (**F**) and ONL (**G**) in various treatments; (**H-J**) The cell density of GCL (**H**), INL (**I**) and ONL (**J**).The data were analyzed by one-way ANOVA with Bonferroni’s post hoc test and unpaired Student’s t-test (2 tailed). ns means no significant difference in indicated two groups.

After treated by different concentrations of TPEN for 24h, the cell viability of aRPE-19 cells were 99.902 ± 3.350, 111.147 ± 5.821, 48.341 ± 2.940, 7.504 ± 0.713, 3.535 ± 0.144 and 2.554 ± 0.461 in groups of 0 μM, 1 μM, 10 μM,100 μM, 1 mM and 10 mM TPEN, respectively (Fig. 3A). No significant difference was found in cell viability of aRPE-19 cells in different groups that containing 1 μM of TPEN (Fig. 3B). The cell viability was 101.980 ± 1.168, 111.359 ± 1.822, 101.522 ± 3.619, 40.702 ± 2.973, 63.734 ± 1.246 and 66.481 ± 3.028 in groups of control, P-NP, PDA-NP, TPEN, TP-NP and TPDA-NP, respectively (Fig. 3C). After aRPE-19 cells were treated by solution or NPs containing 10 μM of TPEN for 24h, its cell viability decreased significantly. Compared with group of TPEN solution, cell viability of aRPE-19 in groups of TP-NP (P < 0.0001) and TPDA-NP (P < 0.0001) was much higher (Fig. 3C), which could be explained by the effect of drug delivery in NPs *in vitro*. As for 661W cells, the viability of cells was 99.993 ± 3.261, 95.201 ± 9.326, 4.837 ± 0.595, 4.313 ± 1.046, 4.064 ± 1.162 and 6.632 ± 0.662 in groups of 0 μM, 1 μM, 10 μM, 100 μM, 1 mM and 10 mM TPEN, respectively (Fig. 3D). Compared with control group, no significant difference was found in cell viability of 661W cells in different groups that containing 1 μM of TPEN (Fig. 3E). As shown in Fig. 3F, the viability of 661W cells was much lower in groups of TPEN solution, TP-NP and TPDA-NP. Cell viability of BV2 cells was remarkably decreased when the concentration of TPEN was higher than 10 μM (Fig. 3G). There was no statistical difference in cell viability of BV2 in different groups, when the concentration of TPEN was 1 μM (Fig. 3H). When the concentration of TPEN was 10 μM, the viability of BV2 cells were 100.013 ± 2.355, 109.234 ± 4.454, 105.531 ± 5.818, 13.664 ± 0.494, 18.218 ± 0.976 and 28.859 ± 3.778 in groups of control, P-NP, PDA-NP, TPEN, TP-NP and TPDA-N, respectively (Fig. 3I). There was a much higher viability of BV2 cells in groups of TP-NP(P = 0.0004) and TPDA-NP(P = 0.0144) when compared with TPEN solution group. Above results indicated that there was no significant toxicity of TPEN in above retinal cells within cultured for 24h, when its concentration was lower than 1 μM. However, an obvious toxicity was indicated when its concentration was higher than 10 μM in aRPE-19, 661W and BV2 cells. In consistent with previous studies, our results also indicated that the toxic concentration of TPEN on cells was lower than 10 μM [36,37]. Human RPE cells swelled when treated by 2 μM TPEN for 24h under SEM, and 0.5 μM TPEN could cause their death when cultured for 48h [37]. However, in this study, we only tested the toxicity of TPEN after cultured for 24h in different concentration. The exact lowest toxic concentration on above cells were not determined. Besides, the functional time of TPEN on cells should also be extended to evaluate its toxicity. To deeply explore the *in vivo* toxic effects of PLGA nanoparticles on different retinal cells, we adopted HE staining to evaluate the thickness of each retinal layer and cell density at 7 days post-treatment. Through the analysis of HE stained retinas, the results clearly demonstrated that various treatment methods did not cause any damage to the retinal structure (Fig. 4A).

During the morphological assessment of the retina, we measured the thickness of each retinal layer. The measurement scope covered not only the entire retina but also extended to each specific sub - layer, including the GCL + RNFL, ONL, OPL, INL, and IPL. As summarized in Table 3 and corroborated by representative histological images (Fig. 4B–G), PLGA nanoparticle administration exhibited no statistically significant alterations in the thickness of any evaluated retinal sublayer compared to baseline controls (p > 0.05). Furthermore, analysis of cellular density within the ONL and INL (Table 4; Fig. 4H–J) confirmed that nanoparticle treatment did not disrupt cytoarchitectural organization, with cell density values remaining consistent across experimental groups.

**Table 3:** Thickness of different retinal layers in various groups (Unit: μm).

	Retina	GCL^a^+ RNFL^b^	IPL^c^	INL^d^	OPL^e^	ONL^f^
CTRL	184.5 ± 7.335	20.62 ± 1.083	38.40 ± 1.898	27.83 ± 0.890	15.86 ± 1.335	42.85 ± 2.749
P-NP	180.5 ± 9.904	18.97 ± 1.649	39.31 ± 0.779	27.99 ± 1.032	15.83 ± 1.524	42.88 ± 2.782
PDA-NP	187.7 ± 7.955	19.9 ± 1.132	40.42 ± 2.575	30.94 ± 0.592	17.19 ± 0.702	43.74 ± 3.824
TPEN	183.4 ± 8.090	21.12 ± 2.739	41.07 ± 2.486	28.94 ± 3.026	16.59 ± 1.033	43.22 ± 0.497
TP-NP	188.0 ± 3.841	20.65 ± 1.215	39.58 ± 1.138	27.99 ± 1.708	15.08 ± 2.065	45.90 ± 1.765
TPDA-NP	184.1 ± 7.270	18.72 ± 0.794	37.76 ± 1.506	30.96 ± 1.240	15.08 ± 2.435	41.95 ± 1.067

a Ganglion Cell Layer.

b Retinal Nerve Fiber Layer.

c Inner Plexiform Layer.

d Inner Nuclear Layer.

e Outer Plexiform Layer.

f Outer Nuclear Layer.

**Table 4:** Cell density in different groups.

	GCL^a^	INL^b^	ONL^c^
CTRL	3414 ± 299	11806 ± 1241	22651 ± 1222
P-NP	3149 ± 299	11517 ± 1558	22940 ± 2539
PDA-NP	3135 ± 276	11025 ± 1292	22935 ± 2235
TPEN	3149 ± 299	11813 ± 1378	22735 ± 1384
TP-NP	3166 ± 282	11095 ± 1782	22585 ± 1799
TPDA-NP	3197 ± 167	10557 ± 1083	21811 ± 1033

a Ganglion Cell Layer.

b Inner Nuclear Layer.

c Outer Nuclear Layer.

### 3.5 TPEN-loaded NPs had superiority over solution in chelating Zn^2+^ after ONC injury

**Fig. 5:**
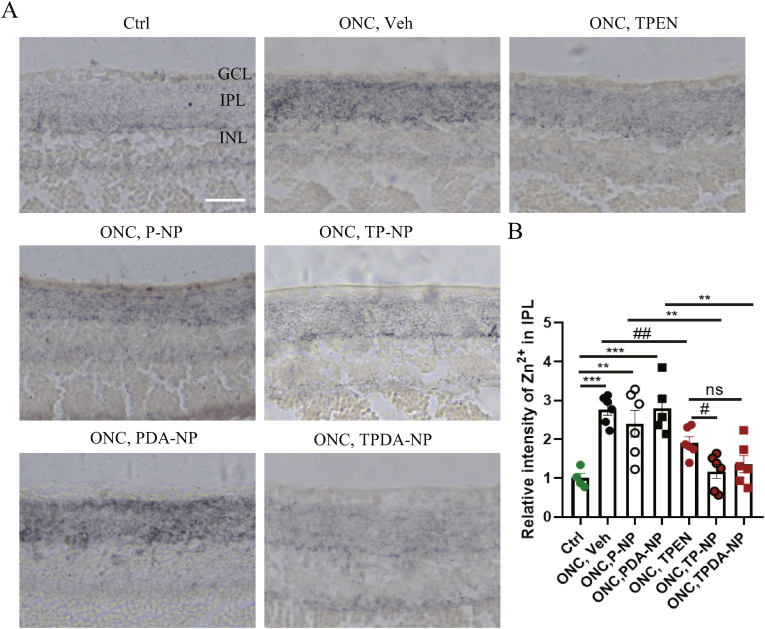
**TPEN-loaded nanoparticles decreased concentration of Zn^2+^ in retinas.** (**A**) Representative images of Zn^2+^ concentration in different groups after 1 day of ONC. Scale bar, 100 μm. (**B**) Bar charts showed the relative intensity of Zn^2+^ in IPL of retinas in different groups. All the data were analyzed by one-way ANOVA with Bonferroni’s post hoc test and unpaired Student’s t-test (2 tailed). ∗∗P＜0.01, ∗∗∗P＜0.001 between indicated two groups. #P＜0.05 between indicated two groups. ##P＜0.01 between indicated two groups.# means unpaired Student’s t-test (2 tailed) between indicated two groups. ns, no significant difference in indicated two groups. Each point shown in the bar charts indicates an individual data point. GCL: Ganglion cell layer; IPL: Inner plexiform layer; INL: Inner nuclear layer.

Since TPEN used as a specific chelator of mobile zinc (Zn^2+^), in order to verify the effect of NPs on chelating Zn^2+^, we conducted autometallography for testing the concentration of Zn^2+^ in retinas. As shown in Fig. 5, the relative intensity of Zn^2+^ in IPL of retinas were 1.000 ± 0.124 (control), 2.767 ± 0.149 (vehicle), 2.401 ± 0.345 (P-NP), 2.793 ± 0.304 (PDA-NP), 1.917 ± 0.150 (TPEN solution), 1.169 ± 0.183 (TP-NP) and 1.363 ± 0.222 (TPDA-NP). Above results indicated that Zn^2+^ significantly increased in retinal IPL after ONC 1 day. Vitreous injection of TPEN solution (0.127 μg TPEN) and TPEN loaded NPs (0.127 μg TPEN) could reduce Zn^2+^ significantly. Besides, compared with TPEN solution, groups of TP-NP (P = 0.0103) and TPDA-NP(P = 0.0658) showed a more obvious effect on reducing Zn^2+^ after ONC, which indicated the superiority of NPs over solution in reducing Zn^2+^ after ONC.

### 3.6 TPEN-loaded NPs had significant superiority over solution in neuroprotection after ONC injury

**Fig. 6:**
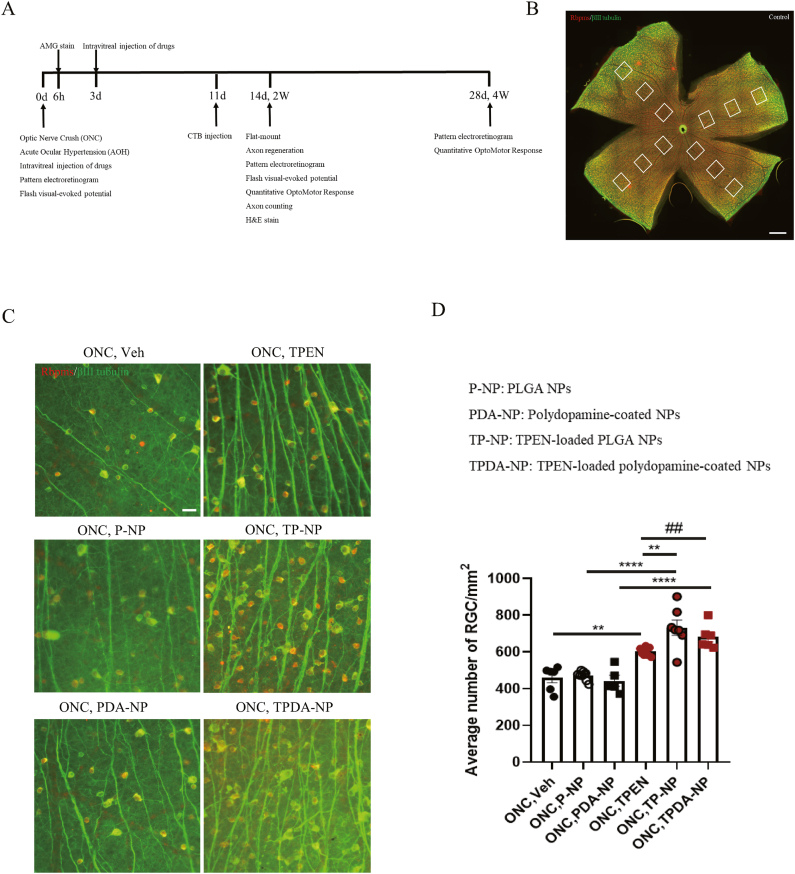
**TPEN-loaded nanoparticles promoted remarkable more RGCs survival. (A)** Schematic of the timeline of performing AOH and ONC injury, intravitreal injection of drugs, and subsequent experiments. (**B**) Image of flat mount of control retina, which showed the 12 areas that were captured from the center, middle, and periphery of the retina for counting number of RGCs. Scale bar, 1 mm. (**C**) Representative images of retinal flat mounts labeled with Rbpms (red) and βⅢ tubulin (green) after 2 weeks of ONC injury in different groups. Scale bar, 20 μm. (**D**) Bar charts showed the average number of both Rbpms and βⅢ tubulin positive RGCs/mm^2^ in the retinas. All the data were analyzed by one-way ANOVA with Bonferroni’s post hoc test and unpaired Student’s t-test (2 tailed). ∗∗P＜0.01, ∗∗∗∗P＜0.0001 between indicated two groups. ##P＜0.01 between indicated two groups. # means unpaired Student’s t-test (2 tailed) between indicated two groups. Each point shown in the bar charts indicates an individual data point. (For interpretation of the references to color in this figure legend, the reader is referred to the web version of this article.)

The schematic timeline of *in vivo* studies is illustrated in Fig. 6A. The ONC model, inducing the severing of RGCs axons, leading to massive RGCs death and regenerative failure, has been extensively employed to investigate RGC survival and nerve regeneration in adult mice [8]. RGCs death serves as a crucial histological indicator in the ONC model and has been pivotal in assessing nerve damage in glaucoma [27]. In order to compare the neuroprotective effects between TPEN-loaded NPs and TPEN solution, retinal flat-mount immunofluorescence was utilized to identify RGCs in retinal flat-mounts [38]. The density of RGCs was determined by counting the average number of cells with both Rbpms and βIII tubulin labeled in the central, middle, and peripheral areas of the retina (Fig. 6B). As shown in Fig. 6C and D, the results indicated that the density of RGCs was 458.833 ± 26.831/mm^2^ (vehicle), 471.778 ± 7.944/mm^2^ (P-NP), 442.800 ± 30.248/mm^2^ (PDA-NP), 604.000 ± 8.218 (TPEN solution, P = 0.0022 VS. vehicle group), 732.286 ± 41.727/mm^2^ (TP-NP, P < 0.0001 VS. P-NP, P = 0.0054 VS. TPEN solution) and 681.500 ± 26.579/mm^2^ (TPDA-NP, P < 0.0001 VS. PDA-NP) in respective ONC groups. These results demonstrated that TPEN could significantly protect RGCs from ONC injury either in the form of a solution or NPs, consistent with previous findings in ONC injury [10,13]. Additionally, both TP-NP (P = 0.005) and TPDA-NP (P = 0.008) exhibited remarkable superiority over TPEN solution on promoting RGCs survival after ONC under the same TPEN dosage, while the neuroprotective effects were similar between TP-NP and TPDA-NP groups. The molecular mechanism of TPEN on neuroprotection may include its anti-inflammatory and anti-apoptosis effect [39,40]. Besides, TPEN could also protect RGCs from ONC injury by inhibiting autophagy and integrated stress response [14,41].

### 3.7 Superiority of TPEN-loaded NPs in axon regeneration post-ONC injury

**Fig. 7:**
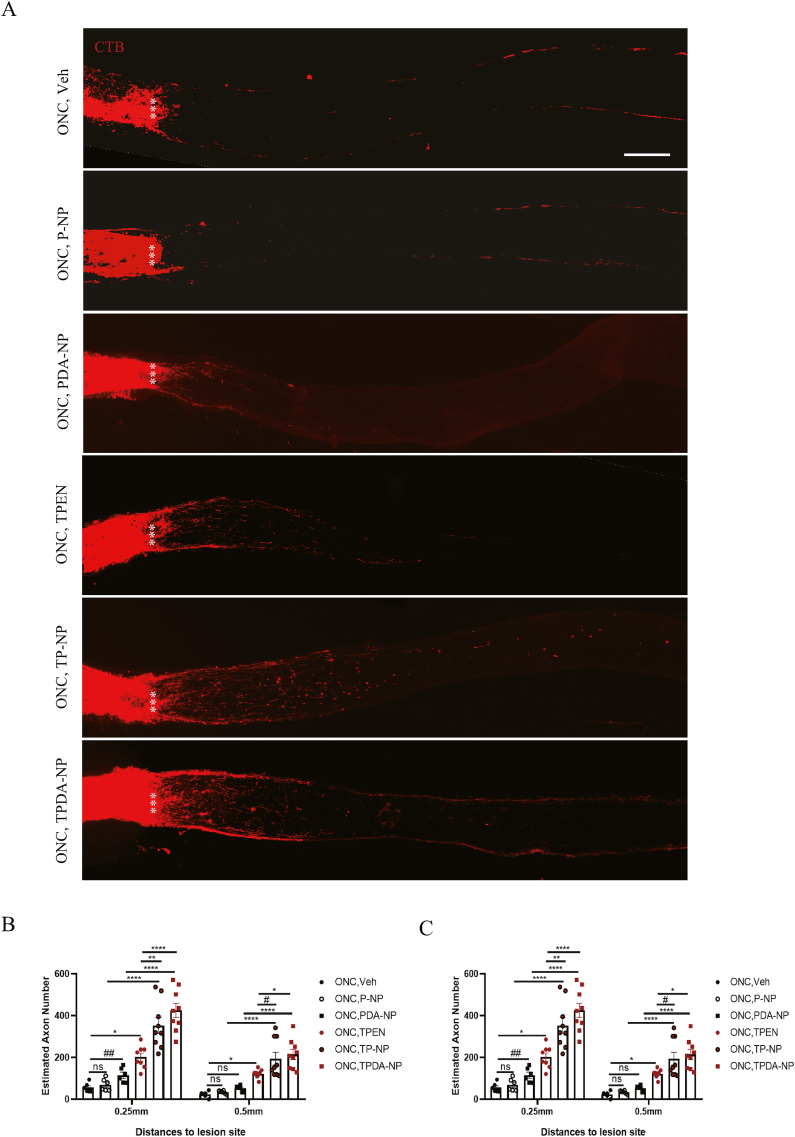
**TPEN-loaded nanoparticles promoted remarkable more axon regeneration.** (**A**) Images of optic nerve wholemounts showed the regenerated axon labeled with CTB (red) after 2 weeks of ONC. Scale bar, 200 μm. (**B**) Quantification of regenerated axons at 0.25 mm and 0.5mm to lesion site in different groups. (**C**) Quantification of regenerated axons at 0.75 mm and 1 mm to lesion site in different groups. All the data were analyzed by one-way ANOVA with Bonferroni’s post hoc test and unpaired Student’s t-test (2 tailed). ∗ P＜0.05, ∗∗P＜0.01, ∗∗∗∗P＜0.0001 between indicated two groups. #P＜0.05, ##P＜0.01 between indicated two groups. # means unpaired Student’s t-test (2 tailed) between indicated two groups. ns, no significance between indicated two groups. Each point shown in the bar charts indicates an individual data point. (For interpretation of the references to color in this figure legend, the reader is referred to the web version of this article.)

In order to compare the axon regenerative effects between TPEN-loaded NPs and TPEN solution, CTB was utilized to identify regenerative axons in optic nerve, due to its high sensitivity and effectiveness in targeting nerve cells [38,42]. The increase of regenerated axons numbers in all testing ONC groups was recorded after applying equal TPEN (0.127 μg), from 55.598 ± 9.232 (vehicle), 69.739 ± 11.861 (P-NP), 115.228 ± 14.335 (PDA-NP, P = 0.0058 VS. vehicle), 202.261 ± 18.985 (TPEN solution, P = 0.0106 VS. vehicle), 353.073 ± 37.522 (TP-NP, P < 0.0001 VS. P-NP) to 425.208 ± 33.432 (TPDA-NP, P < 0.0001 VS. PDA-NP), at a distance of 0.25 mm from the lesion site (Fig. 7A and B). The results related to axon regeneration demonstrated that compared with the vehicle, P-NP, PDA-NP groups, TPEN solution, TP-NP and TPDA-NP treatments significantly promoted axonal regeneration after ONC injury (Fig. 7A–C), confirming the efficacy of TPEN in axon regeneration [10]. Furthermore, compared with TPEN solution, both TP-NP (P = 0.0025) and TPDA-NP (P < 0.0001) significantly enhanced axon regeneration after ONC injury, although the effects on axon regeneration were comparable between TP-NP and TPDA-NP groups (Fig. 7A–C). Above results demonstrated that applying NPs for TPEN-loaded delivery could increase its efficiency in treating ONC injury, which confirmed the therapeutic benefits from sustained NPs release system [[43], [44], [45]]. Surprisingly, compared with the vehicle-treated group, the PDA-NP group significantly increased the regenerated axons after ONC (P = 0.0058 VS. vehicle group). The observed effect of PDA-NP on axon regeneration may be attributed its elimination function of reactive oxygen species (ROS) [46]. However, previous results (Fig. 6C and D) demonstrated that PDA-NP had no superior over the vehicle group in RGCs survival. The differential effects of PDA on neuroprotection and axon regeneration may arise from distinct mechanisms in these processes. Despite the observation that nearly every treatment triggering axon regeneration enhances RGC survival, implying a potential connection between cell survival and axon outgrowth pathways [47], sustaining RGC survival alone proves insufficient for axon regeneration. Notably, the pro-survival effects of ATF3 and Bcl2 overexpression do not translate into optic nerve regeneration [48,49]. Additionally, brain-derived neurotrophic factor (BDNF) and rapamycin, while demonstrating neuroprotective effects, paradoxically diminishes axon restoration [50,51]. The possible mechanism of TPEN in promoting axon regeneration may be explained by its regulation on growth-related genes and autophagy [10,41]. The limited regeneration of axons of TPEN may be improved by extending experimental period or combinatory treatment such as TPEN add gene therapy [10].

### 3.8 Enhanced therapeutic effects through increased frequency of vitreous TPEN solution injection post-ONC injury

**Fig. 8:**
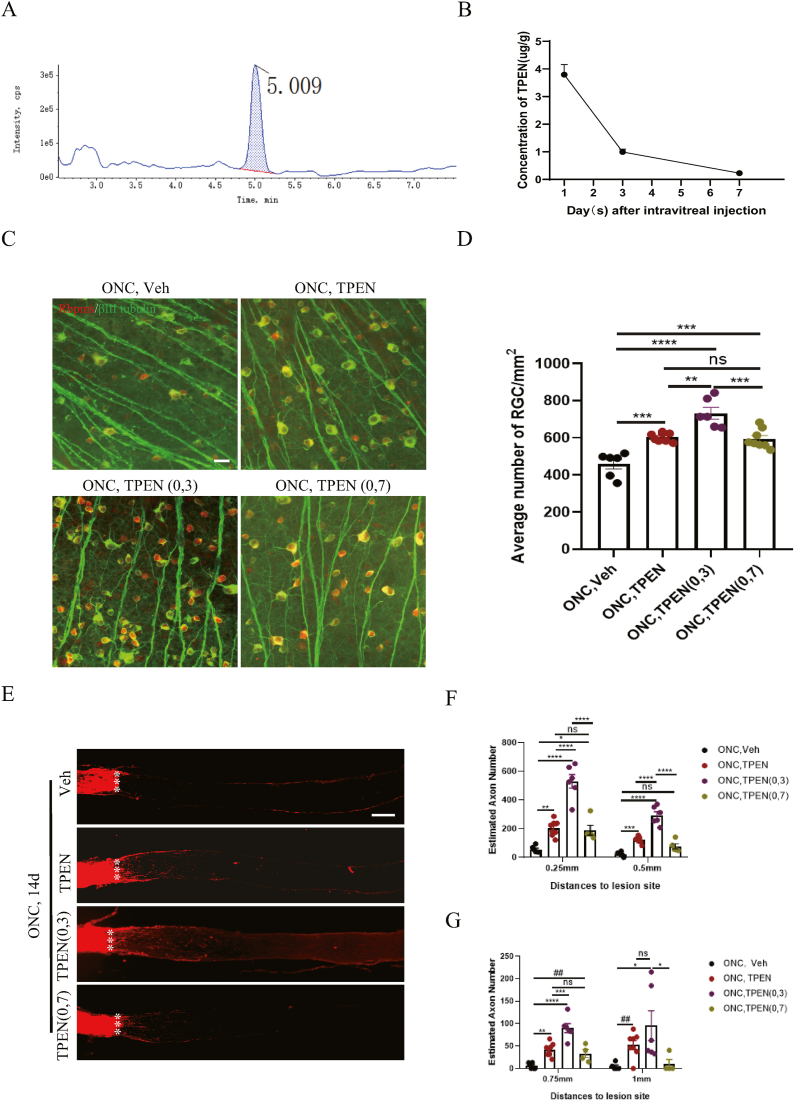
**Multiple treatment of TPEN solution promoted significantly more RGCs survival and axon regeneration.** (**A**) Detection time of TPEN in retinas by UPLC-ESI-MS/MS. (**B**) Change dynamic of concentration of TPEN in retinas after different days of ONC. (**C**) Representative images of flat-mounted retinas showing the both Rbpms and βⅢ tubulin positive RGCs in different groups. Scale bar, 20 μm. (**D**) Quantification of average number of RGCs/mm^2^ after 2 weeks of ONC in different groups. (**E**) Representative images of optic nerve wholemounts showed the regeneration of axon labeled with CTB after 2 weeks of ONC. Scale bar, 200 μm. (**F-G**) Number of regenerated axons at different distances to lesion. TPEN (0,3) means vitreous injections of TPEN (100 μM) were applied immediately and 3 days after ONC. TPEN (0,7) means vitreous injections of TPEN (100 μM) were applied immediately and 7 days after ONC. Data were analyzed by one-way ANOVA with Bonferroni’s post hoc test and unpaired Student’s t-test (2 tailed). ns, no significance between indicated two groups. ∗P＜0.05, ∗∗P＜0.01, ∗∗∗P＜0.001, ∗∗∗∗P＜0.0001 between indicated two groups. ##P＜0.01 between indicated two groups. # means unpaired Student’s t-test (2 tailed) between indicated two groups. Each point indicates an individual data point.

The limited therapeutic impact of TPEN may stem from its low concentration in retinal tissue, owing to ocular barriers that hinder drug absorption [52]. Initially, we analyzed the concentration of TPEN in mice retinas after ONC by UPLC-ESI-MS/MS at various time points. Results indicated that the retention time of TPEN was approximately 5.0 min in UPLC-ESI-MS/MS (Fig. 8A). The TPEN concentration was 3.793 ± 0.366 μg/g, 0.993 ± 0.101 μg/g and 0.227 ± 0.043 μg/g after 1, 3 and 7 days of ONC, respectively (Fig. 8B). To enhance the therapeutic effect of TPEN solution on RGCs survival and axon regeneration, we increased the injection frequency to prolong the duration of a relatively high concentration of the drug [22]. Vitreous injection of TPEN was administered twice after ONC injury. To determine the optimal time for multiple injections, vitreous injections of TPEN (100 μM) were applied immediately and 3 or 7 days after ONC. Results indicated that adding one more injection of TPEN solution on day 7 (594.500 ± 18.2851/mm^2^) after ONC conferred no superiority over a single injection (604.000 ± 8.218/mm^2^) in RGCs protection (Fig. 8C and D). However, adding one more injection of TPEN solution on day 3 (732.833 ± 31.790/mm^2^) after ONC remarkably promoted RGCs survival compared to a single injection (P = 0.001) (Fig. 8C and D). Similar results were observed for axon regeneration after ONC injury. One more injection of TPEN solution on day 3 (530.220 ± 47.135 at a distance of 0.25 mm from the lesion site) remarkably promoted more axon regeneration than one more injection of TPEN solution on day 7 (189.751 ± 34.230 at a distance of 0.25 mm from the lesion site) or a single injection (202.261 ± 18.985 at a distance of 0.25 mm from lesion site) after ONC immediately (Fig. 8E–G). The distinct effects on RGCs protection and axon regeneration were attributed to the different time points adding one injection, possibly due to the limited reaction time of RGCs to TPEN. Our above results indicated that increased injection frequency enhanced therapeutic benefits for RGCs protection and axon regeneration, consistent with other studies, albeit with different injection frequencies and timings [22,53]. However, more efficient outcomes may be achievable by extending the experimental observation time, necessitating further studies [54].

### 3.9 Comparable effects of half and the same dosage of TPEN-NPs in promoting RGCs survival and axon regeneration post-ONC

**Fig. 9:**
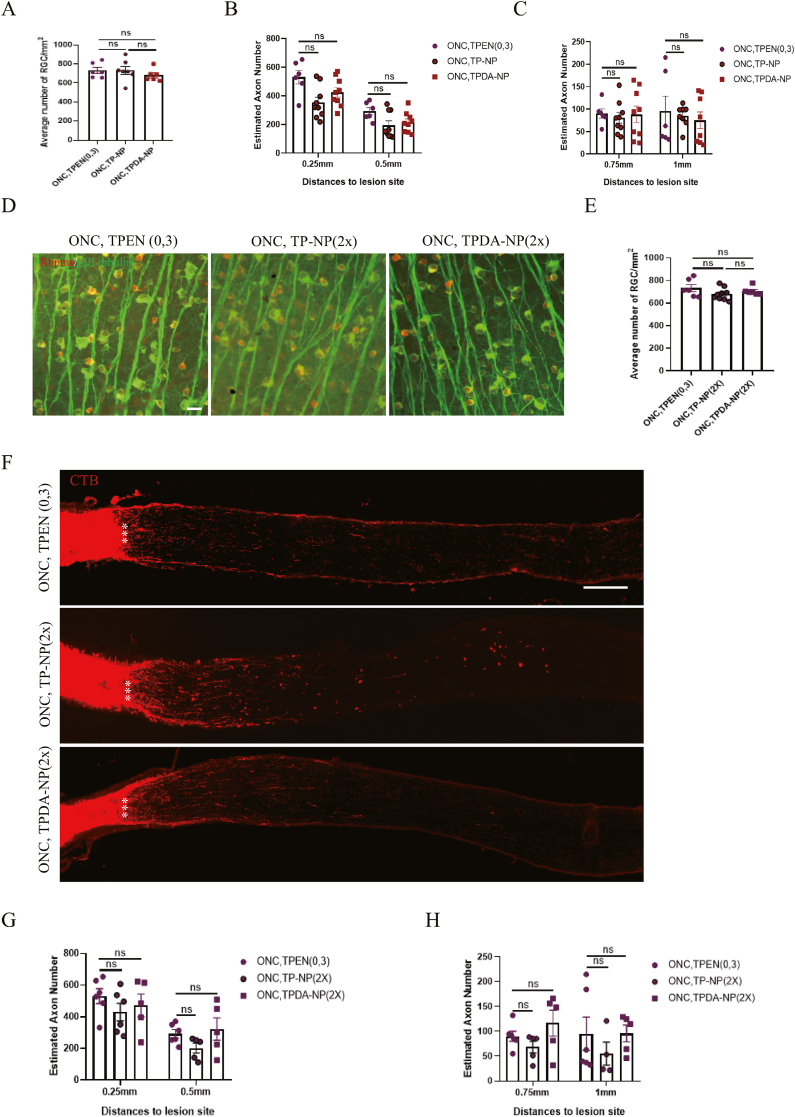
**TPEN-loaded nanoparticle had no superiority over multiple treatment of TPEN in the same or half dosage on RGCs survival and axon regeneration.** (**A**) Bar charts showed the average number of both Rbpms and βⅢ tubulin positive RGCs/mm^2^ in the retinas. (**B-C**) Quantification of regeneration of fibers at different distances to lesion site in each group. (**D**) Representative images of flat-mounted retinas showing the both Rbpms and βⅢ tubulin positive RGCs in different groups. Scale bar, 20 μm. (**E**) Quantification of average number of RGCs/mm^2^ after 2 weeks of ONC in different groups. (**F**) Representative images of optic nerve wholemounts showed the regeneration of axon labeled with CTB after 2 weeks of ONC. Scale bar, 200 μm.(**G-H**) Number of regenerated axons at different distances to lesion site. Data were analyzed by one-way ANOVA with Bonferroni’s post hoc test. ns, no significance between indicated two groups. Each point shown in the charts indicates an individual data point.

Above results demonstrated that both twice injections of TPEN solution and a single injection of TPEN-loaded NPs significantly enhanced RGCs survival and axon regeneration compared to a single TPEN solution injection after ONC injury. We subsequently examined the comparative effects of twice TPEN solution injections (0.254 μg TPEN) and a single injection of TPEN-loaded NPs (0.127 μg TPEN) on RGC survival and axon regeneration. The data indicated that a single injection of TPEN-loaded NPs injection (with the same TPEN dosage as a single TPEN solution injection) exhibited similar effects in promoting RGCs survival (Fig. 9A) and axon regeneration (Fig. 9B and C) compared to twice TPEN solution injections after ONC injury. These findings suggested that the delivery system of TPEN-loaded NPs could achieve a comparable therapeutic effect with reduced vitreous injection frequency and halved TPEN dosage. However, when the TPEN dosage in TPEN-loaded NPs was equivalent to the twice injection of TPEN solution (0.254 μg TPEN), the effects on RGCs survival (Fig. 9D and E) and axon regeneration after ONC (Fig. 9F–H) were similar. The quality of TPEN (0.254 μg) was the same in twice injection of TPEN solution and single injection of nanoparticles, frequency of injection was reduced in TPEN-loaded nanoparticle, which decreased the damage of vitreous injection, and showed the necessity and advantages of encapsulation of TPEN by NPs. Above results may be attributed to the limited number of surviving RGCs capable of responding to TPEN. Half the dosage of TPEN in nanoparticles (0.127 μg TPEN) proved sufficient for the remaining RGCs to absorb, in comparison to twice the solution injection (0.254 μg TPEN). The utilization of fluorescent nano-systems for drug tracking could potentially provide a solution for evaluating the functional range and concentration of drugs [55].

### 3.10 TPEN facilitates recovery of visual function post-ONC injury

**Fig. 10:**
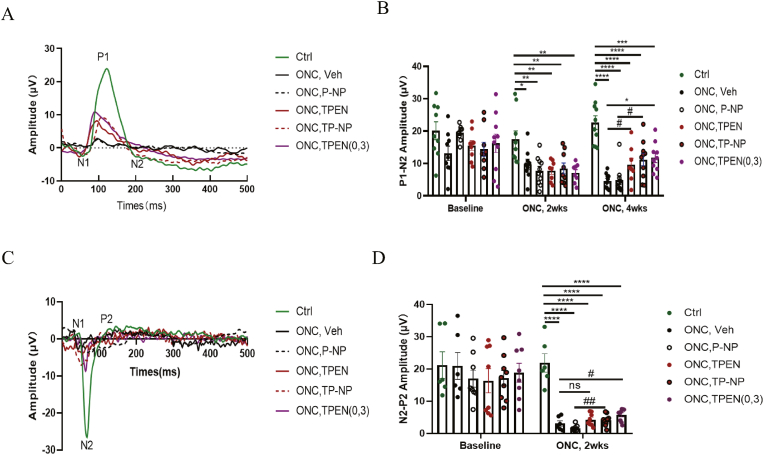
**TPEN-loaded nanoparticle had comparable therapeutic effect with TPEN in the same dosage in preserving visual function.** (**A**) Representative pictures of pERG waveform performed 4 weeks after ONC injury of different groups. The representative points of N1, P1 and N2 of control group were showed in the picture. (**B**) Bar plots showed the quantitative analysis of pERG P1–N2 amplitudes before ONC (Baseline), 2 weeks and 4 weeks after ONC of different groups. (**C**) Representative images of waveform in different groups of f-VEP. The points of P1, N2 and P2 in the waveform were showed in control group. (**D**) Bar graph showed the quantitative analysis of N2-P2 amplitude of f-VEP in different groups before and after 2 weeks of ONC injury. All data were displayed as mean ± SEM and were analyzed using one-way ANOVA followed by Dunnett’s multiple comparisons test or unpaired Student’s t test (2 tailed). ∗ P < 0.05, ∗∗P < 0.01, ∗∗∗P＜0.001, ∗∗∗∗P＜0.0001 between indicated two groups. #P＜0.05, ##P＜0.01 between indicated two groups. # means unpaired Student’s t-test (2 tailed) between indicated two groups. ns, no significance between indicated two groups. Each point shown in the statistic charts indicates an individual data point.

Preceding evidence suggested that alterations in inner retinal circuitry precede RGCs degeneration and optic nerve atrophy in experimental glaucoma models [56]. Besides, functional changes, particularly electrophysiological alterations are considered to better reflect early stages of neurodegeneration compared to assessing RGCs death [57]. To evaluate the electrophysiological functional changes in the retina and optic nerve, we measured the pERG response (P50-N95 amplitude) reflecting RGCs functional changes and the f-VEP (N2–P2 wave) representing optic nerve and visual pathway function at various time points post-ONC injury [58,59]. As shown in Fig. 10, at baseline, there were no significant difference between the groups in P1-N2 amplitude and N2–P2 wave. Fig. 10A and B revealed that after 2 weeks of ONC injury, single TPEN solution injection (7.81 ± 1.12 μV), TP-NP (8.48 ± 1.66 μV) and twice TPEN solution injection (7.09 ± 1.04 μV) had no discernible effect on restoring the P1-N2 amplitude compared to vehicle (9.80 ± 1.57 μV) and P-NP (7.73 ± 1.39 μV) groups. However, after 4 weeks of ONC injury, single TPEN solution injection (9.64 ± 1.94 μV), TP-NP (11.19 ± 1.88 μV) and twice TPEN solution injection (11.70 ± 1.45 μV) significantly increased the P1-N2 amplitude compared to vehicle (4.59 ± 0.73 μV, P = 0.018), P-NP (4.90 ± 1.32 μV, P = 0.013) and vehicle (4.59 ± 0.73 μV, P = 0.0316) groups respectively, indicating a protective effect of TPEN after ONC on the function of retinal ganglion cells. Fig. 10C and D showed a significantly lower N2-P2 amplitude of f-VEP after 2 weeks of ONC in vehicle (3.06 ± 0.84 μV), P-NP (1.59 ± 0.34 μV), TPEN (4.29 ± 0.69 μV), TP-NP (4.13 ± 0.60 μV) and TPEN (0,3) (5.75 ± 0.64 μV) groups, compare to control groups. Both TPEN solution and TP-NP had an effect on restoring N2-P2 amplitudes. Furthermore, TP-NP and TPEN (0,3) groups significantly increased N2-P2 amplitudes compared with P-NP group (P = 0.003) and vehicle group (P = 0.023), indicating that TP-NP and twice TPEN solution injection had a better effect on the recovery of N2-P2 amplitudes than single TPEN solution injection. These results revealed that TPEN slightly promoted the recovery of visual function in the damaged retina and optic nerve after ONC. The limited recovery of visual function after ONC by TPEN or TP-NP may result from the limited survival number of RGCs and regenerated axon by them after ONC injury. The duration of nerve regeneration observation and the concentration of drug used may also contribute to the relatively mild repairment of mice visual function.

### 3.11 TPEN nanoparticles provided enhanced protection for RGCs and axons following AOH compared to solution

**Fig. 11:**
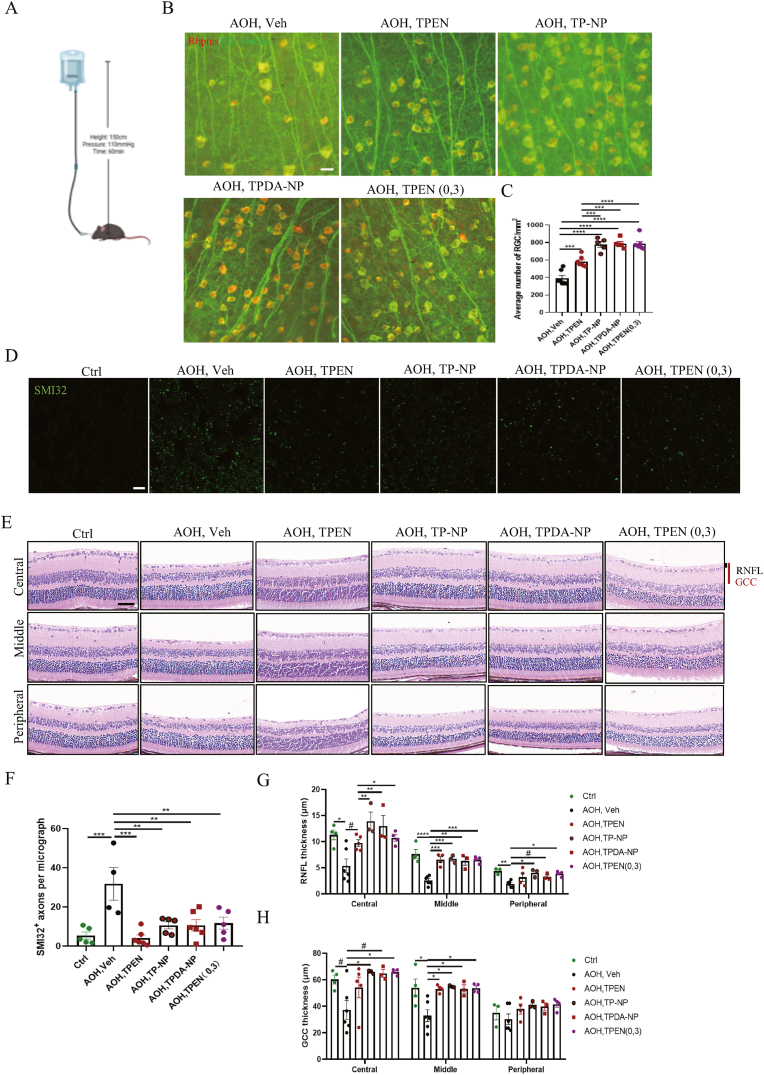
**TPEN-loaded nanoparticles preserved significantly more RGCs and axon after AOH than solution.** (**A**) A diagram of AOH injury modeling. (**B**) Representative images of retinal flat mounts labeled with Rbpms (red) and βⅢ tubulin (green) after 2 weeks of AOH injury in different groups. Scale bar, 20 μm. (**C**) Bar plots showed the quantitative analysis of RGCs/mm^2^ in 2 weeks after AOH of different groups. (**D**) Representative images of SMI32^+^ axons in different groups. Scale bar, 40 μm. (**E**) Representative HE staining images of paraffin‐embedded retinal sections from different groups. Scale bar, 80 μm. (**F**) Bar graph showed the number of SMI32^+^ axons per micrograph in different groups after 2 weeks of AOH injury. (**G-H**) Quantification of the thickness of the RNFL and GCC in central, middle, and peripheral retina regions from different groups. All data were displayed as mean ± SEM and were analyzed using one-way ANOVA followed by Dunnett’s multiple comparisons test or unpaired Student’s t test (2 tailed). ∗P < 0.05, ∗∗P＜0.01, ∗∗∗P＜0.001, ∗∗∗∗P＜0.0001 between indicated two groups. #P＜0.05 between indicated two groups. # means unpaired Student’s t-test (2 tailed) between indicated two groups. Each point shown in the statistic charts indicates an individual data point. (For interpretation of the references to color in this figure legend, the reader is referred to the web version of this article.)

The precise molecular mechanisms underlying glaucoma remain elusive, with various animal models being utilized for research in this field [60]. AOH serves as an extensively utilized model for studying glaucoma due to its key characteristic of acute glaucoma (Fig. 11A). Several studies have utilized the AOH model to investigate therapeutic approaches for protecting RGCs and axons in glaucoma [[61], [62], [63]]. In this study, we employed the AOH model to investigate the protective effects of TPEN and TPEN-loaded NPs on RGCs, with a focus on quantifying the number of RGCs in different groups. Results demonstrated that TPEN significantly enhanced the protection of RGCs from AOH injury, whether in the form of a solution (581.143 ± 22.462/mm^2^, P = 0.0002) or NPs (P < 0.0001) compare to vehicle group (Fig. 11 B and C). What's more, both TP-NP (780.000 ± 32.941/mm^2^, P = 0.0004), TPDA-NP (789.200 ± 24.270/mm^2^, P = 0.0002) and twice vitreous injection of TPEN solution (784.143 ± 27.213/mm^2^, P < 0.0001) exhibited remarkable superiority over single injection of TPEN solution in promoting RGCs survival after AOH. Although the neuroprotective effects were similar between the two types of NPs (P = 0.999). To visualize and evaluate damaged axons 2 weeks after AOH in different groups, SMI32, a marker for dephosphorylated neurofilaments of damaged axons, was utilized. SMI32 is most abundant in the early stages of lesion formation, and according to experimental data, only a proportion of these damaged axons will undergo axonal transection [64,65]. Fig. 11D and F demonstrated that the number of SMI32^+^ axons per micrograph significantly increased after AOH (31.750 ± 8.347, P = 0.0004 VS. control group) injury compared with the control group (5.367 ± 1.868). Single TPEN solution injection (4.083 ± 1.637, P = 0.0001 VS. vehicle group), TP-NP (10.600 ± 1.824, P = 0.0048 VS. vehicle group), TPDA-NP (10.639 ± 2.877, P = 0.0033 VS. vehicle group) and twice vitreous injection of TPEN solution (11.733 ± 3.096, P = 0.0082 VS. vehicle group) significantly downregulated the number of SMI32^+^ axons after AOH, though NPs showed no obvious superiority over the solution in axon protection. Additionally, H&E stain was employed to estimate the thickness of RNFL and ganglion cell complex (GCC). Significant thinning of the inner retina, particularly the RNFL and GCC was indicated in vehicle-treated AOH group (Fig.11E and G-H). The thickness of RNFL in the control, vehicle, TPEN, TP-NP, TPDA-NP and TPEN (0,3) groups were 11.308 ± 0.950 μm, 5.375 ± 1.314 μm, 9.753 ± 0.622 μm, 13.906 ± 1.724 μm, 12.989 ± 2.015 μm and 10.692 ± 0.620 μm in the center of sections. The thickness of RNFL and GCC was thinner in the middle and peripheral area than the center. H&E staining of retinal sections indicated that TPEN solution and TPEN-loaded NPs could restore the thickness of RNFL and GCC after AOH injury, although TPEN-loaded NPs did not exhibit superior efficiency compared to the solution. Though we have evaluated the RGCs and axons protective effect of TPEN on AOH glaucomatous model, the efficacy of TPEN and NPs on visual function and the deeper molecular mechanism need to be further explored. Besides, the therapeutic effect of TPEN on other glaucomatous animal model, such as transgenic animal models and normal tension glaucoma, should also be testified in the further studies.

## 4 Conclusions

In this study, we successfully developed a biodegradable nanoparticles system based on PLGA for *in vivo* delivery of TPEN. The cumulative findings suggest that TPEN nanoparticles, formulated with PLGA and PDA, can serve as an effective drug release system, thereby enhancing therapeutic efficacy in glaucoma models of ONC and AOH. TPEN-loaded NPs achieved a comparable therapeutic effect while requiring a reduced frequency of vitreous injection and lower dosage compared with solution. The establishment of this NPs delivering system and the methodologies employed herein present a promising strategy for glaucoma treatment, offering potential applications in the development and pre-clinical assessment of neuroprotective and neuro-regenerative therapies.

## CRediT authorship contribution statement

**Caiqing Wu:** Writing – review & editing, Writing – original draft, Project administration, Methodology, Investigation, Data curation, Conceptualization. **Haitao Zhang:** Writing – review & editing, Writing – original draft, Methodology, Conceptualization. **Yuze Chen:** Investigation, Data curation. **Yangjiani Li:** Methodology, Investigation, Data curation. **Weiwei Jin:** Investigation, Formal analysis. **Jinpeng Yang:** Investigation, Formal analysis, Data curation. **Yehong Zhuo:** Writing – review & editing, Project administration, Conceptualization. **Ziyu Gao:** Writing – review & editing, Writing – original draft, Project administration, Methodology, Conceptualization. **Xiaohong Hu:** Validation, Supervision, Project administration, Methodology, Conceptualization. **Yiqing Li:** Writing – review & editing, Supervision, Resources, Project administration, Funding acquisition, Conceptualization.

## Declaration of competing interest

The authors declare that they have no known competing financial interests or personal relationships that could have appeared to influence the work reported in this paper.

## Data Availability

Data will be made available on request.
